# Mucosal microbiota and gene expression are associated with long-term remission after discontinuation of adalimumab in ulcerative colitis

**DOI:** 10.1038/s41598-020-76175-2

**Published:** 2020-11-05

**Authors:** Toshiharu Sakurai, Hiroki Nishiyama, Kazuko Sakai, Marco A. De Velasco, Tomoyuki Nagai, Yoriaki Komeda, Hiroshi Kashida, Akiyoshi Okada, Isao Kawai, Kazuto Nishio, Hiroyuki Ogata, Masatoshi Kudo

**Affiliations:** 1 Department of Gastroenterology and Hepatology, Kindai University Faculty of Medicine, 377-2 Ohno-Higashi, Osaka-Sayama, Osaka, 589-8511 Japan; 2 Institute for Chemical Research, Kyoto University, Uji, 611-0011 Japan; 3 Department of Genome Biology, Kindai University Faculty of Medicine, Osaka, Japan; 4 Wakakusa Daiich Hospital, Higashi-Osaka, Japan; 5 Ootori Stomach and Intestine Hospital, Sakai, Japan

**Keywords:** Biomarkers, Translational research

## Abstract

Given that sustained remission is the ultimate treatment goal in the management of patients with ulcerative colitis (UC), the decision to stop anti-tumor necrosis factor (anti-TNF) treatment in UC patients is difficult. The aim of this study was to evaluate mucosal microbiota and gene expression profiles associated with long-term remission after discontinuation of anti-TNF therapy. In nine UC patients who received anti-TNF therapy for 6 months, microbiota isolated from uninflamed mucosae and gene expression in inflamed and uninflamed mucosae were investigated at week 0 and at week 24. At treatment initiation, *Fusobacterium* sp. and *Veillonella dispar* were over-represented in the relapse group compared with the non-relapse group. After treatment, *Dorea* sp. and *Lachnospira* sp. were over-represented in the non-relapse group. In the relapse group only, a significant shift in gut bacterial community composition was found between week 0 and week 24. Gene expression of *ALIX* (*PDCD6IP*) and *SLC9A3* was significantly higher in the non-relapse group than in the relapse group. Lastly, we used machine learning methods to identify relevant gene signatures associated with sustained remission. Statistical analyses of microbiota and expression profiles revealed differences between UC patients who did or did not keep remission after the discontinuation of TNF inhibitors.

**Trial registration**: UMIN000020785: Evaluation of adalimumab therapy in mesalazine-resistant or -intolerant ulcerative colitis; an observational study (EARLY study).

## Introduction

The advent of anti-tumor necrosis factor (anti-TNF) agents has revolutionized the treatment of inflammatory bowel disease (IBD), which comprises ulcerative colitis (UC) and Crohn’s disease (CD). Anti-TNF agents are able to induce mucosal healing and decrease the risk of hospitalization and surgery, which has resulted in better long-term outcomes in IBD patients^1–3^. Patients who achieve remission with anti-TNF agents are often treated for many years if they are able tolerate the treatment. Therefore, the number of IBD patients treated with anti-TNF agents is increasing, resulting in high costs^4^. Long-term treatment with anti-TNF agents is generally considered to be safe, though these drugs can sometimes cause side effects such as infections, skin and joint problems, and some malignancies, especially when administered in combination with immunomodulators^5–8^.

In the clinical setting, some IBD patients hope to discontinue anti-TNF treatment due to the risk of side effects and personal preference. However, concerns related to treatment withdrawal include risk of flare, possible loss of efficacy if the drug has to be restarted, risk of infusion reactions or other adverse events during re-treatment, and worries about losing medical treatment options^9–12^. When making decisions about elective discontinuation of anti-TNF therapy, it is essential to be able to identify patients who are more likely to achieve long-lasting remission; this will also provide a novel foundation for personalized medicine.

Machine learning, a method of data analysis that automates analytical model building, contributes to the integration of multiple risk factors into a predictive tool^13^ and has become an increasingly popular tool for medical researchers to predict cancer susceptibility, recurrence and survivability^14^. In the field of IBD, with the availability of genome wide association study (GWAS) data, machine learning has been successfully used^15,16^.

A major burden experienced by IBD patients is that the disease follows a relapsing–remitting course over many years. In this pilot study, we treated mesalazine-resistant or -intolerant UC patients with TNF inhibitors for 6 months and examined the gut mucosal microbiota and gene expression levels at week 0 and week 24 of anti-TNF therapy to explore factors related to long-term remission after withdrawal of TNF inhibitors. We applied machine learning to whole transcriptome data to develop a proof of concept model that could identify UC patients with long-term remission after discontinuation of anti-TNF therapy.

## Results

### Patient characteristics

In total, nine patients with UC who had no previous treatment with anti-TNF agents or immunomodulators were treated with adalimumab (ADA) for 6 months then stopped (Table 1). The median follow-up time of the patients was 32.5 months (IQR 23–44). All patients achieved clinical remission at week 28, while Mayo endoscopic subscore was 1 at week 24. In four out of nine patients (Patient #4–7), ADA induced remission that was maintained until week 72 (non-relapse group). In contrast, the relapse group was defined as patients who relapsed before week 72 (Patient #1–3 and #8–9). Retreatment with the same anti-TNF agent ADA was successful in three out of four patients (Patient #1, 2, 6 and 9), whilst one patient (Patient #6) did not respond to retreatment with ADA probably since the serum trough ADA level had decreased from 3.5 to 2.0 μg/mL after the retreatment.

**Table 1:** Characteristic of patients.

	Age	Sex	Type	Duration (years)	Smoking habit	Week 0		Week 24		Week 28	Time to relapse (mo)	Group	Present therapy	Follow-up period (months)
						Endo. subscore	Mayo score	Endo. subscore	Serum ADA level	SCCAI				
#1	23	m	E3	2	Never	2	6	1	> 10	2	1	Relapse	ADA	23
#2	17	m	E2	0	Never	3	8	1	6.8	2	6	Relapse	ADA	25
#3	49	m	E3	25	Active	2	8	1	2.9	2	6	Relapse	GLM	24
#4	25	m	E3	4	Never	2	8	1	> 10	2	14	Non	5-ASA	40
#5	14	m	E3	1	Never	3	9	1	1.2	2	14	Non	5-ASA	28
#6	19	f	E3	2	Ex-smoker	2	6	1	3.5	1	18	Non	GLM	44
#7	37	m	E2	7	Never	3	9	1	2.3	2	26	Non	AZA	36
#8	46	f	E3	4	Never	3	9	1	1.9	2	1	Relapse	IFX	41
#9	14	f	E3	0	Never	2	5	1	0.5	2	2	Relapse	ADA	29

Serum ADA level (μg/mL).

*m* male, *f* female, *Endo. Subscore* mayo endoscopic subscore, *ADA* adalimumab, *SCCAI* simple clinical colitis activity index, *mo* months, *Non* the non-relapse group, *AZA* azathioprine, *GLM* golimumab, *IFX* infliximab, *Follow-up period* follow-up period after initiation of anti-TNF-α therapy.

### Gut bacterial communities are different between patient groups at treatment baseline and between time points in the non-relapse group

16S rRNA gene amplicon analyses were conducted on the gut bacterial communities of UC patients who did or did not maintain remission until week 72 (i.e. the non-relapse and relapse groups, respectively), at two time points: week 0 and week 24, i.e., the time point just before anti-TNF therapy was withdrawn (Table 1). Due to inadequate sample quality or failure to perform biopsies, Patient #1 (at post-treatment), Patient #2 (at both treatment baseline and post-treatment), and Patient #4 (at treatment baseline) were not assessed. The analyses resulted in 6418 operational taxonomic units (OTUs), backed by 1,538,484 merged reads (Table 2).

**Table 2:** Amount of sequences derived from biopsies.

Time point of treatment	Patient group	Patient #	Pairs of raw reads	Merged reads	Reads in OTUs
Treatment baseline	Relapse	1	87,925	82,231	72,268
		3	114,584	107,584	90,415
		8	589,051	557,459	458,963
		9	243,332	230,916	181,417
	No relapse	5	100,868	57,224	44,572
		6	59,695	55,381	45,911
		7	139,849	130,278	90,919
Post-treatment	Relapse	3	157,325	150,378	127,160
		8	32,964	27,495	18,646
		9	100,914	75,526	64,074
	No relapse	4	127,363	105,692	81,083
		5	200,621	156,633	134,519
		6	60,609	56,400	48,346
		7	96,892	91,538	80,191
Total			2,111,992	1,884,735	1,538,484

Comparisons of richness and alpha diversity were performed between patient groups for each time point (two comparisons) and between time points for each patient group (two comparisons). OTU richness showed no clear difference between the compared sample groups (non-parametric two-sample *t*-test, *P* > 0.05; Fig. S1, Table S1). Shannon’s diversity index also demonstrated no statistical differences between patient groups or time points (non-parametric two-sample *t*-test, *P* > 0.05) (data not shown).

**Figure 1:**
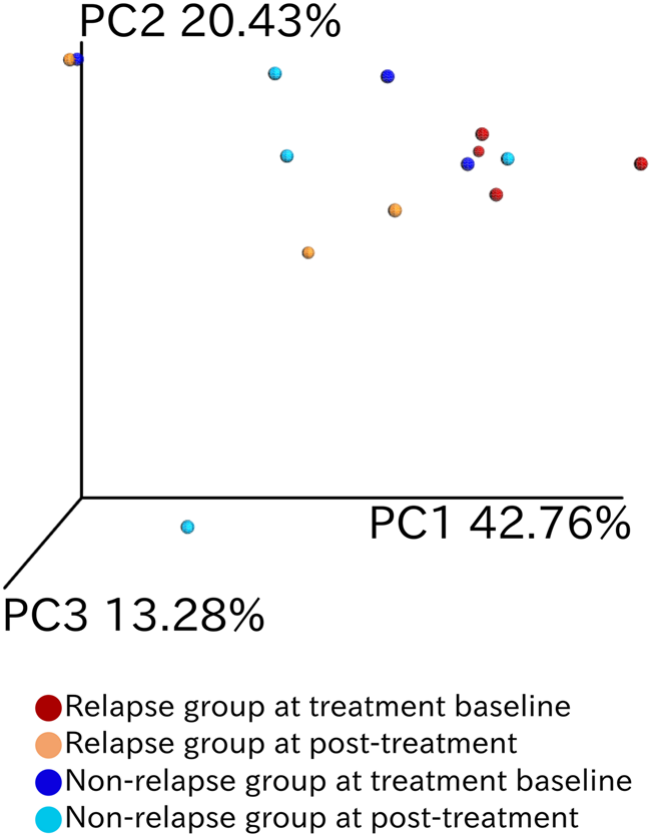
Compositional difference between gut bacterial communities in each patient at different treatment time points. Principal coordinate analysis was conducted on pairwise weighted UniFrac distances between samples. The color codes are as follows: red, relapse group at week 0; orange, relapse group at week 24; blue, non-relapse group at week 0; and turquoise, non-relapse group at week 24.

Samples from the non-relapse and relapse groups at week 0 were discriminated from each other in the principal coordinate analysis (PCoA) based on weighted UniFrac distances (Fig. 1). The bacterial composition was found to be statistically different between these two groups (Adonis test, *P* = 0.029, effect size = 0.294). Furthermore, the bacterial community composition of the relapse group at week 24 changed significantly from that at week 0 (Adonis test, *P* = 0.029, effect size = 0.340).

### Bacterial relative abundance differs across patient groups and time points

**Figure 2:**
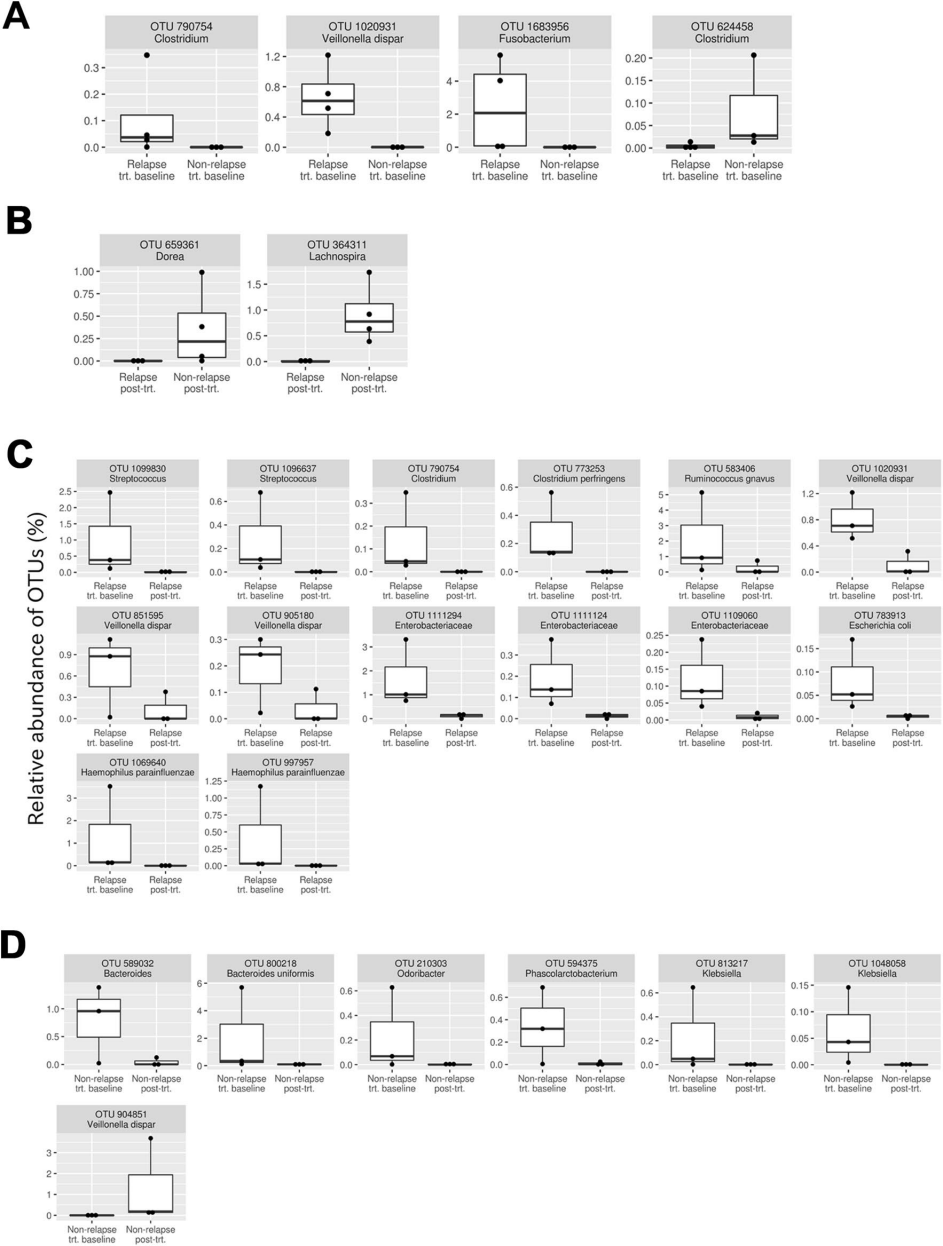
Bacterial OTUs that demonstrated differential abundances in the following comparisons (false discovery rate < 0.05). (**A**) Non-relapse group at week 0 *vs.* relapse group at week 0 of anti-TNF therapy. (**B**) Non-relapse group at week 24 *vs.* relapse group at week 24 of anti-TNF therapy. (**C**) Relapse group at week 0 *vs.* relapse group at week 24 of anti-TNF therapy. (**D**) Non-relapse group at week 0 *vs.* non-relapse group at week 24 of anti-TNF therapy.

To further characterize the dissimilarity of bacterial composition among the samples, the differential abundance of each bacterial species was tested with DESeq2 (Fig. 2). Comparisons were first performed between the non-relapse and relapse groups at each treatment time point to identify candidate bacteria associated with the difference in the period of drug-induced remission. At week 0, four OTUs displayed differential abundance between the two patient groups (Fig. 2A). Three of these were over-represented in the relapse group: *Clostridium* sp. (one OTU), *Fusobacterium* sp. (one OTU), and *Veillonella dispar* (one OTU). The other OTU was over-represented in the non-relapse group, *Clostridium* sp. (one OTU). At week 24, two OTUs, *Dorea* sp. (one OTU) and *Lachnospira* sp. (one OTU) were significantly over-represented in the non-relapse group (Fig. 2B).

Next, comparisons were performed between samples at week 0 and week 24. In the relapse group, 14 OTUs were over-represented in samples obtained at week 0 (Fig. 2C): *Streptococcus* spp. (two OTUs), *Clostridium* sp. (one OTU), *Clostridium perfringens* (one OTU), *Ruminococcus gnavus* (one OTU), *Veillonella dispar* (three OTUs), Enterobacteriaceae spp. (three OTUs), *Escherichia coli* (one OTU), and *Haemophilus parainfluenzae* (two OTUs). In the non-relapse group, seven OTUs were over-represented in samples obtained at week 0 (Fig. 2D); specifically, *Bacteroides* sp. (one OTU), *Bacteroides uniformis* (one OTU), *Odoribacter* sp. (one OTU), *Phascolarctobacterium* sp. (one OTU), and *Klebsiella* spp. (two OTUs) while *Veillonella dispar* (one OTU) was over-represented in samples obtained at week 24.

### Specific gene expression signatures are associated with maintenance of remission after discontinuation of TNF inhibitors

**Figure 3:**
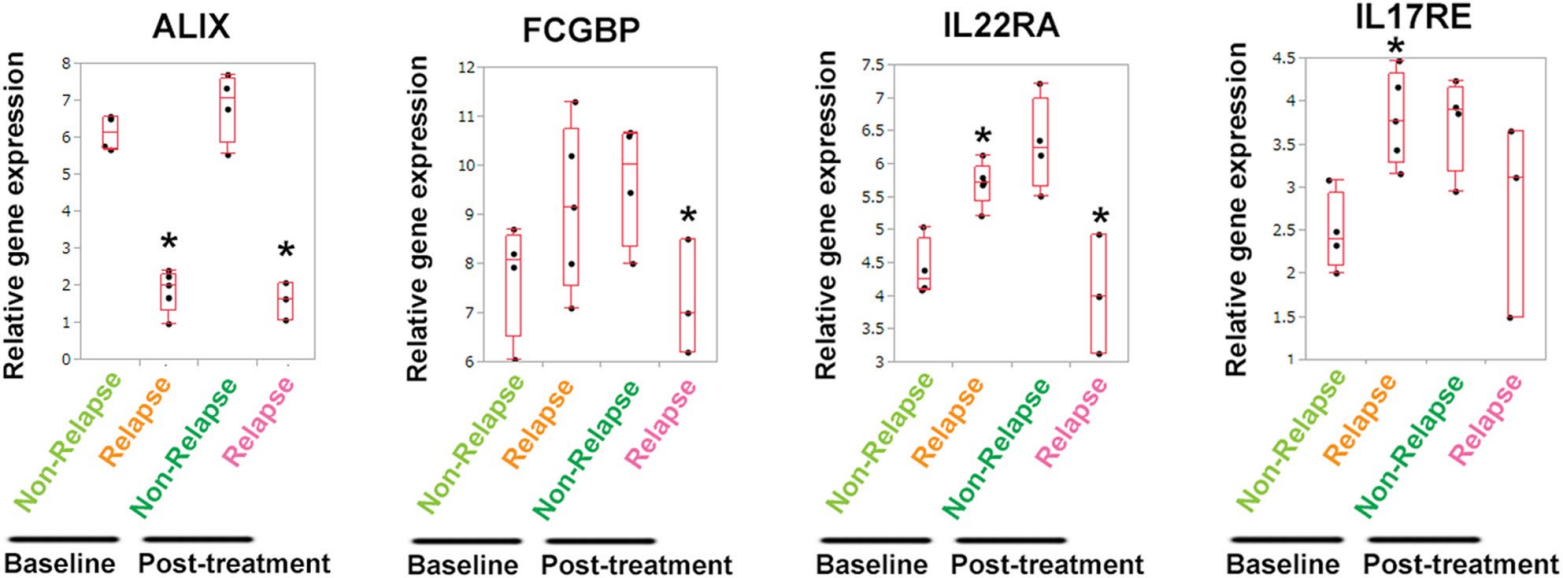
Differentially expressed genes in rectal mucosae of UC patients at week 0 and 24 of anti-TNF therapy. (**A**) Gene expression signatures in inflamed mucosae (rectum) between the non-relapse group (Non-relapse) and relapse group (Relapse). Upregulated or downregulated genes in the non-relapse group are shown compared with those in the relapse group. *P < 0.05 compared with the non-relapse group at week 0 (Baseline) or at week 24 (Post-treatment).

We compared the gene expression signatures in rectal inflamed mucosae in the non-relapse and relapse UC patient groups at week 0 and week 24. Compared with the non-relapse group, the relapse group demonstrated significant downregulation of *ALIX* (*PDCD6IP*) and *SOX10* at week 0, and *ALIX*, *FCGBP* (a typical mucus component), *IL22RA*, and *LGR6* at week 24 (Fig. 3, Figs. S2A, S3A, and Table S1). In contrast, the significantly upregulated genes in the relapse group included *IL17RE* at week 0, and *HSPB2*, *HSPB6*, and *MAPK4* at week 24 (Figs. S2B, S3B). When comparisons were performed between samples at week 0 and week 24, differential expression patterns were found in some genes. In the relapse group, the expression levels of *IL22RA* and *SLC9A3* were significantly lower than those in the non-relapse group at week 24, whereas these were significantly upregulated in the relapse group at week 0 (Fig. 3 and Fig. S3A). In contrast, inverse associations were found in the expression levels of *HSPB2*, *HSPB6* and *MAPK4*; compared with the non-relapse group, the relapse group showed downregulation of these genes at week 0 and upregulation at week 24 (Fig. S3B).

Whole transcriptomic sequencing was performed in the uninflamed mucosae of UC patients. Compared with the non-relapse group, significantly downregulated genes in the relapse group included *ALIX* and *AK1* at week 0, and *ALIX*, *BCL2,* and *LGR6* at week 24 (Fig. S4A and Table S1). In contrast, significantly upregulated genes in the relapse group included *ETV5* and *HAAO* at week 0 (Fig. S4B).

### Use of machine learning to characterize informative gene features from whole transcriptome data associated with maintaining remission after discontinuation of anti-TNF therapy

**Figure 4:**
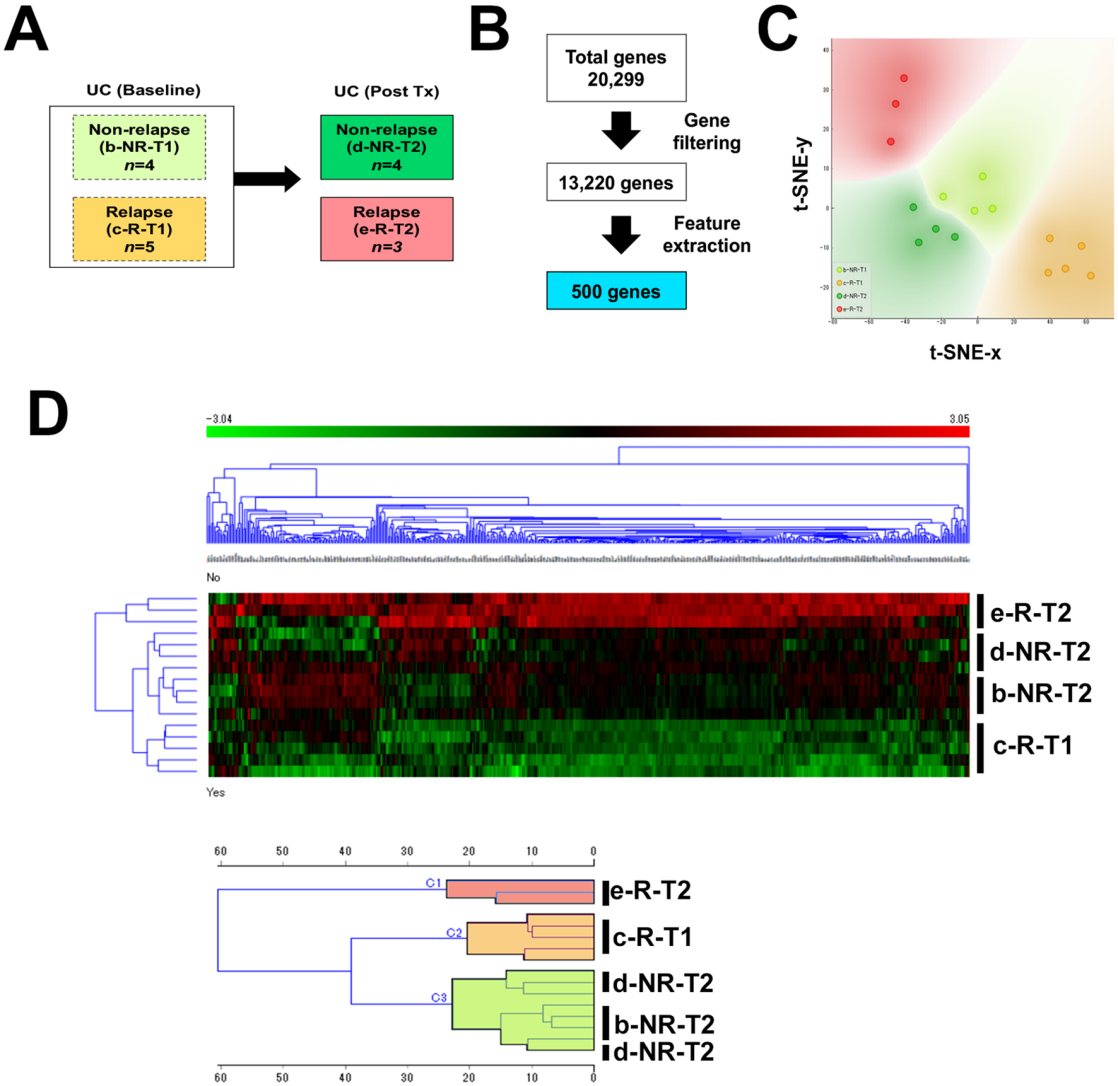
(**A**) Schema for comparative analysis of the transcriptome between groups. (**B**) Summary workflow for feature extraction for supervised analysis. (**C**) t-SNE visualization of patient cluster assignments. (**D**) Clustering analysis of extracted genes in UC patients at week 0 and 24. Heatmap shows unsupervised hierarchical clustering of selected genes using average linkage and Euclidean distance and dendrogram clustering used Ward’s linkage and Euclidean distance.

We next employed machine learning approaches in order to gain more insights into transcriptomic landscape of UC patients that maintain long-term remission after discontinuation of TNF therapy. Gene expression datasets were classified into non-relapse at week 0 (b-NR-T1), relapse at week 0 (c-R-T1), non-relapse at week 24 (d-NR-T2), and relapse at week 24 (e-R-T2) groups (Fig. 4A). We used a supervised approach to select 500 informative genes associated with each target group from a total pool of 20,299 genes (Fig. 4B). We were able to identify three distinct clusters: cluster 1 was comprised of all e-R-T2 patients; cluster 2 contained all c-R-T1; and cluster 3 included all of b-NR-T1 and d-NR-T2, indicating a distinct molecular signature for patients that maintained remission status (Fig. 4C,D).

**Figure 5:**
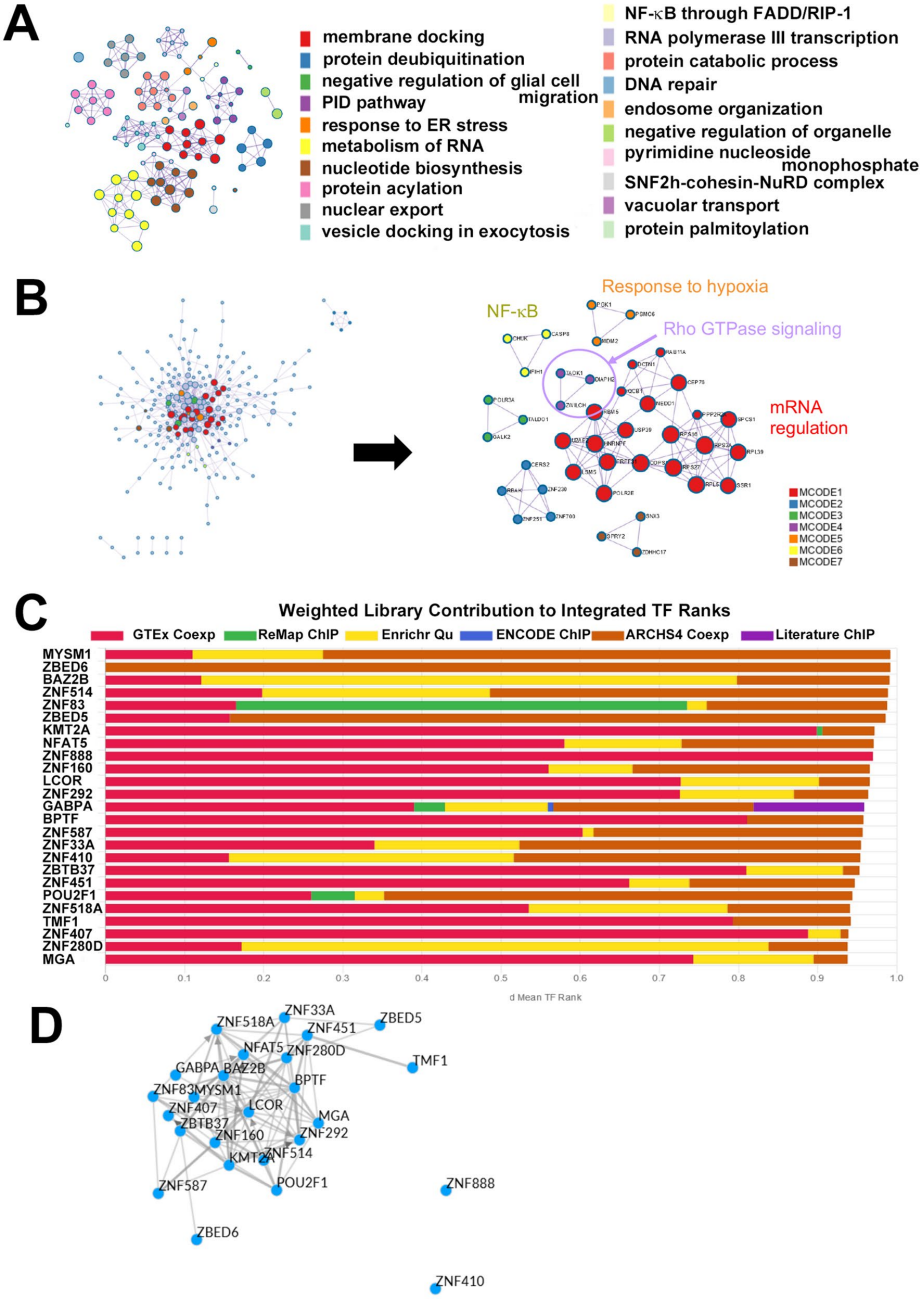
Functional characterization of 385 genes that were upregulated at week 24 in the relapse group compared with the non-relapse group (**A**) Enriched ontology clusters and memberships. (**B**) Protein–protein interaction (PPI) network and Molecular Complex Detection (MCODE) components. (**C**) Transcription factor analysis. Top 25 putative transcription factors based on ChEA3. Selected transcription factors were assembled from various sources and were determined by the average integrated rank. (**D**) Visualization of transcription factor-transcription factor local co-regulatory networks. Edges between transcription factors are defined by evidence from the ChEA3 libraries.

To infer molecular regulatory mechanisms related to long-term remission in UC patients, we identified statistically enriched terms from 385 genes that are upregulated at week 24 in the relapse group compared with the non-relapse group using GSEA (Fig. S5A). These genes were associated with various GO memberships including membrane docking, response to endoplasmic reticulum stress, metabolism of RNA, and NF-κB activation (Fig. 5A and Fig. S5B). Protein–protein interaction using MCODE identified mRNA regulation, Rho GTPase signaling, response to hypoxia and NF-κB signaling as key biologically relevant components (Fig. 5B and Fig. S6A). Next, we identified putative transcription factors regulating the 385 genes by ChiP-X Enrichment Analysis (ChEA3). The top 25 transcription factors, based on cumulative weighted mean transcription factor ranks of integrated libraries are shown in Fig. 5C,D. Of these *MYSM1* was to top ranked transcription factor and had 38 overlapping genes, however *GABPA*, *ZNF83*, and *KMT2A* had 150, 71 and 70 overlapping genes, respectively. We further queried for putative transcription factors from the published literature and *cAMP-responsive element modulator* (*CREM*) was identified as the top ranked putative transcription factor with 167 overlapping genes (Fig. S6B).

### Use of machine learning to model prediction of long-term remission after discontinuation of anti-TNF therapy

**Figure 6:**
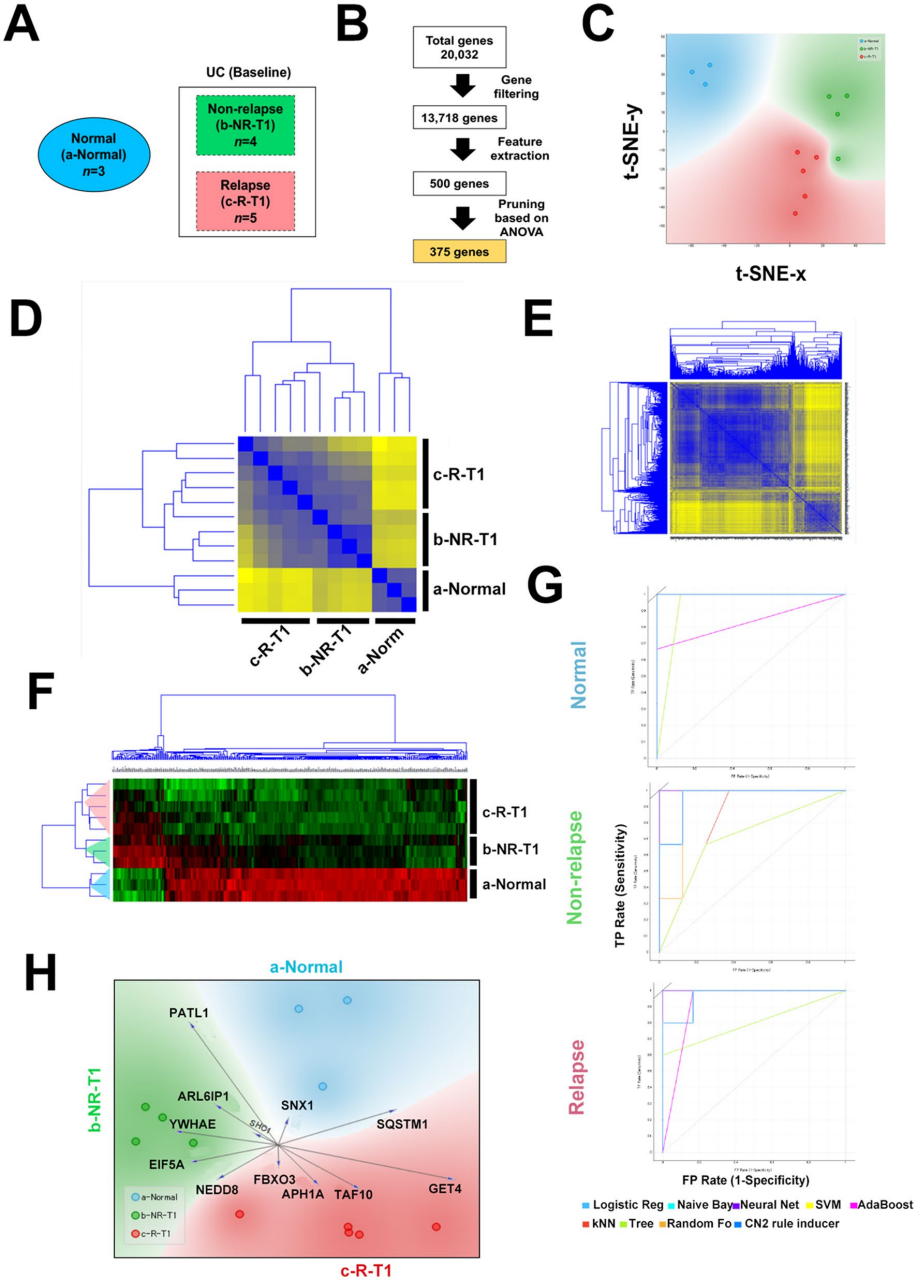
(**A**) Schema for comparative analysis of UC patients for prediction modeling. (**B**) Summary of feature extraction for prediction modeling. (**C**) t-SNE visualization in 2 dimensions for 500 genes. Correlation matrix heatmap showing the Euclidean distance between patient classes (**D**) and the 500 selected genes (**E**). (**F**) Clustering analysis of 375 genes selected for prediction modeling. Heatmap shows unsupervised hierarchical clustering using average linkage and Euclidean distance (**G**) Receiver operating characteristics of the prediction model according to class. (**H**) Multivariate visualization using FreeViz indicates the 12 most informative genes associated with each class.

We next aimed to develop a model that utilized transcriptomic data to predict long-term remission. For this we used machine learning to identify informative genes that could discriminate healthy normal colonic mucosa from that of UC patients and furthermore differentiate the non-relapse and the relapse groups (Fig. 6A). After gene filtering and feature extraction, 500 genes were selected as candidate classification features (Fig. 6B). Overall grouping by these classifiers showed a distinct grouping between the non-relapse and the relapse groups (Fig. 6C,D). Correlation analysis of genes revealed two main clusters (Fig. 6E). An unsupervised approach yielded similar patterns of stratification with some overlap between the classes (Fig. S7). To refine the model, we performed pruning and features were reduced to 375 genes (Fig. 6B), resulting in clear stratification of patients (Fig. 6F). Given the small sample size, we were unable to split into training and test sets, however, we used this cohort as a training model to test this our approach of refined feature selection to develop a prediction model. Using cross validation sampling, we tested different learning algorithms. A summary of the performance is on Fig. S8A,B, overall logistic regression, naïve Bayes, neural network and support vector machine (SVM) had the best performance over all classes (Fig. 6G, Fig. S8A,B). Lastly, we extracted a 12 gene signature based on the most informative genes associated with each classification which might be able to predict long-term remission after withdrawal of anti-TNF therapy (Fig. 6H and Fig. S8C).

## Discussion

When considering elective anti-TNF discontinuation, it is of great value in the decision-making process to be able to predict which patients maintain remission. Factors associated with risk of relapse after discontinuation of anti-TNF therapy include the absence of mucosal healing, the lack of immunosuppressant maintenance treatment after anti-TNF is stopped, younger age, and the discontinuation of anti-TNF inhibitors due to adverse events^9,17^. However, no clinical factors have been associated with maintenance of remission at a satisfactory level of evidence. Low trough levels at the time of anti-TNF discontinuation were associated with a lower relapse risk in Crohn’s disease patients but not in UC patients^11,12^. Consistently, our results indicated that the trough level cannot be incorporated into clinical practice in UC.

Two out of the three OTUs that were over-represented in the relapse group at treatment baseline (Fig. 2A) were potentially pro-inflammatory: *Fusobacterium* sp. (one OTU) and *Veillonella dispar* (one OTU). Specific species of *Fusobacterium*, such as *F. nucleatum* and *F. varium*, were reported to be associated with the promotion of intestinal inflammation^18,19^. The population of *V. dispar* was shown to be significantly larger in patients with severe disease compared to those with mild disease in a large-scale study of gut bacterial communities affected by IBD^20^. In addition, *V. dispar* produces hydrogen sulfide, which promotes intestinal inflammation^21,22^. At week 24, two OTUs were over-represented in the non-relapse group (Fig. 2B): *Dorea* sp. (one OTU) and *Lachnospira* sp. (one OTU). Members of both bacterial genera are known to produce short-chain fatty acids, which serve as a carbon source for intestinal epithelial cells and also induce regulatory T cells^23,24^ that are beneficial for maintaining gut homeostasis^25–27^. Overall, these findings suggest that observation of the gut microbiome at week 0 or 24 may help to predict the sustainability of remission after initial recovery achieved by anti-TNF therapy in UC patients.

OTUs showing differential abundances between the two treatment time points were also identified in each patient group (Fig. 2C,D). The shift of bacterial communities induced by treatment in the relapse group was accompanied by a larger number of differentially abundant OTUs in comparison with the non-relapse group. Specifically, 14 OTUs showed differential abundance between week 0 and 24 in the relapse group, while only seven OTUs were differentially abundant in the non-relapse group.

In the relapse group, several OTUs potentially corresponding to pro-inflammatory bacteria showed a significant decrease in relative abundance after treatment. One such OTU was *Clostridium perfringens*, which is composed of various toxin-producing strains associated with enteric diseases such as enteritis necroticans and diarrhea^28^. However, because *C. perfringens* is known to be present in healthy humans, it remains unknown whether the decrease of this OTU is causally related to treatment-induced remission. Other OTUs were identified as *R. gnavus*, *V. dispar*, and *H. parainfluenza* (one, three, and two OTUs, respectively). These bacterial species have been associated with a higher severity of IBD^20^. It should be noted that *R. gnavus* was recently found to secrete inflammatory glucorhamnan polysaccharides, which promote production of the pro-inflammatory cytokine TNF by dendritic cells^29^. Also, *V. dispar*, as mentioned previously, secretes hydrogen sulfide^21^.

In contrast to the relapse group, the bacterial community shift in the non-relapse group showed a more intricate trend. In agreement with the achieved clinical remission, two of the OTUs that showed a significant decrease in relative abundance after treatment corresponded to *Klebsiella* spp. Strains of *Klebsiella* have been shown to induce colitis in genetically susceptible mice^30^. However, the changes of other OTUs were not consistent with the achieved clinical remission. Specifically, four OTUs (*Bacteroides* sp., *B. uniformis*, *Odoribacter* sp., and *Phascolarctobacterium* sp.) that decreased their relative abundance were assigned to genera/species known to produce short chain fatty acids, which are considered to be beneficial for intestinal homeostasis^31–33^. Furthermore, the OTU that showed a significant increase in relative abundance during treatment corresponded to *V. dispar*, which produces hydrogen sulfide^21^.

Here we identified several genes that may be linked to long-term remission. ALIX (ALG-2-interacting protein X, also known as programmed cell death 6 interacting protein: PDCD6IP) functions within the endosomal sorting complex required for transport (ESCRT) pathway and is involved in endocytosis, multivesicular body biogenesis, membrane repair, apoptosis, and maintenance of tight junction integrity^34,35^. Interestingly, ALIX protein expression is decreased in severe colitis^36^. The IL22 receptor (IL22RA) is present in many epithelial tissues, including LGR5-positive intestinal stem cells, and the IL22/IL22RA axis is involved in stem cell proliferation, epithelial defense, and wound healing^37^. LGR6, a LGR5 homologue, acts as a Wnt receptor component that mediates Wnt signal enhancement by soluble R-spondin proteins^38^. Thus, LGR6 is likely to be required for epithelial homeostasis and may be critical for controlling IBD. A recent study reported that congenital sodium diarrhea due to the *SLC9A3* mutation is a cause of neonatal diarrhea secondary to dysfunction of the Na + /H + antiporter 3 in the intestine^39^. Given that the expression of *ALIX*, *IL22RA*, *LGR6*, and *SLC9A3* was downregulated after anti-TNF therapy in the relapse group, these genes might prevent flare-ups of UC. In contrast, heat shock proteins (*HSPB2*, *HSPB6*, and *HSPB7*), *IL17RE*, and *ITGA7* (integrin alpha 7) were upregulated in the relapse group. Heat shock protein and IL17 signaling pathways are reported to be implicated in refractory IBD^40–42^. Dysregulation of NF-кB, Rho GTPase, and hypoxia pathways can lead to inflammation in IBD^43,44^, which is consistent with our findings (Fig. 5A,B). Moreover, we identified CREM as putative transcription factor that may be responsible for several genes enriched in relapsed cases and published reports indicate that CREM is key regulator of enteric inflammation^45^. These gene expression signatures might help predict relapsing and refractory clinical courses in UC patients.

The major limitation of this work is the low number of patient samples. Still, our findings offer valuable data and provides a proof of concept approach for prediction modelling that will serve as the foundation for further training and validation. It is also important to note that patients in this cohort received different colonoscopy preparation agents and mechanical bowel preparations, which have been shown to influence intestinal microbial composition^46^.

Overall, our results showed that gut bacterial communities differed more between patient groups at week 0 than week 24 of anti-TNF therapy. We also showed that gut bacterial communities in each patient group demonstrated a shift in expression pattern resulting from treatment. Previous studies have reported differences in gut microbiota and gene expression between to anti-TNF therapy responders and non-responders^47–49^. Herein, we examined differences in gut bacterial communities and gene signatures among responders to anti-TNF therapy who did or did not achieve remission up to 72 weeks. Given that IBD follows a relapsing–remitting course, the proper management of IBD patients requires the ability to predict the clinical course and optimize therapeutic strategy. Thus, mucosal microbiota and gene expression signatures might be indicative of disease behavior and therefore have the potential to be of clinical relevance in the future.

## Methods

### Study design and patient population

This was a multi-center observational pilot study performed at Kindai University, Wakakusa Daiichi Hospital, and Ootori Stomach and Intestine Hospital. UC patients with no history of therapy with anti-TNF agents or immunomodulators treated with ADA between January 2016 and November 2017 were enrolled in this study. Six months after the initiation of ADA treatment, 9 patients achieved clinical remission and treatment was stopped. Patients were prospectively followed by IBD experts at regular outpatient clinic appointments. Clinical, biochemical, endoscopic, and radiological evaluation were performed during follow-up at each physician’s discretion. Endoscopy was performed at the start of TNF inhibitors (week 0) and at week 24, and biopsies were taken from the inflamed regions (rectum) and endoscopically uninflamed regions (ileum). Endoscopic scoring was performed in a blinded fashion by three investigators (Y.K., T.N., and H.K.). Trough ADA levels in the serum at week 24 were measured using the Humira/ADA ELISA Kit for humans (Alpha Diagnostics Intl. Inc., San Antonio, Texas).

Clinical remission was defined as a Simple Clinical Colitis Activity Index (SCCAI) of ≤ 2^50^. Relapse was defined as a SCCAI ≥ 5 or the need for (re)treatment^51^. The non-relapse group was defined as patients who maintained remission until week 72 (Patient #4–7), while the relapse group was defined as patients who relapsed before week 72 (Patient #1–3 and #8–9). This study was approved by the ethical committee of the Kindai University Faculty of Medicine and other relevant institutions (#28–224). All methods were performed in accordance with the relevant guidelines and regulations. All patients provided informed consent prior to their enrollment in the study.

### Microbiome and transcriptome analysis

DNA and RNA were extracted simultaneously from the same biopsy samples using the AllPrep DNA/RNA Mini Kit (Qiagen). DNA derived from uninflamed mucosae samples were subjected to 16S rRNA gene amplicon sequencing. Briefly, 16S rRNA amplicon sequences were processed based on previously described methods^52,53^, with additional modifications. Gene expression was evaluated using the AmpliSeq Transcriptome Human Gene Expression Kit (Thermo Fisher Scientific) according to the manufacturer’s instructions. Details of bioinformatics and supervised machine learning analysis methods are described in the supplemental methods.

To further minimize the inaccurate identification of differentially abundant OTUs, those which passed our criteria based on false discovery rate were examined in the following way. For each of these OTUs, presence was checked in the samples in the high-abundance group. If any of the samples contained no reads for the OTU, we did not consider this OTU to be differentially represented. In addition, if the highest relative abundance of an OTU in the low-abundance group was larger than its relative abundances in the samples from the high-abundance group for more than half of the cases, the OTU was not considered to be differentially represented.

### Prediction modeling

For prediction modeling gene expression data was filtered and preprocessed as previously described. Informative genes were selected by ANOVA and were pruned to the top 75% (375 genes). Classification was performed using various learner models tested using cross validation sampling (10 folds) and performance was evaluated by classification accuracy and recall.

## Supplementary information

Supplementary Information

Supplementary Information

Supplementary Information

Supplementary Information

Supplementary Information

Supplementary Information

Supplementary Information

Supplementary Information

Supplementary Information

Supplementary Information

Supplementary Information

## Data Availability

Raw 16S rRNA gene amplicon sequences were deposited to DNA Data Bank of Japan/Sequence Read Archive (DDBJ/DRA) under the accession number DRA009655. The merged reads are available on the following site: ftp://ftp.genome.jp/pub/db/community/microbiome_kindai/.
