# Supplementary figures and images for: Mucosal microbiota and gene expression are associated with long-term remission after discontinuation of adalimumab in ulcerative colitis
Source: Sci Rep. 2020 Nov 5;10:19186. doi: 10.1038/s41598-020-76175-2 (PMC7644643; doi:10.1038/s41598-020-76175-2)

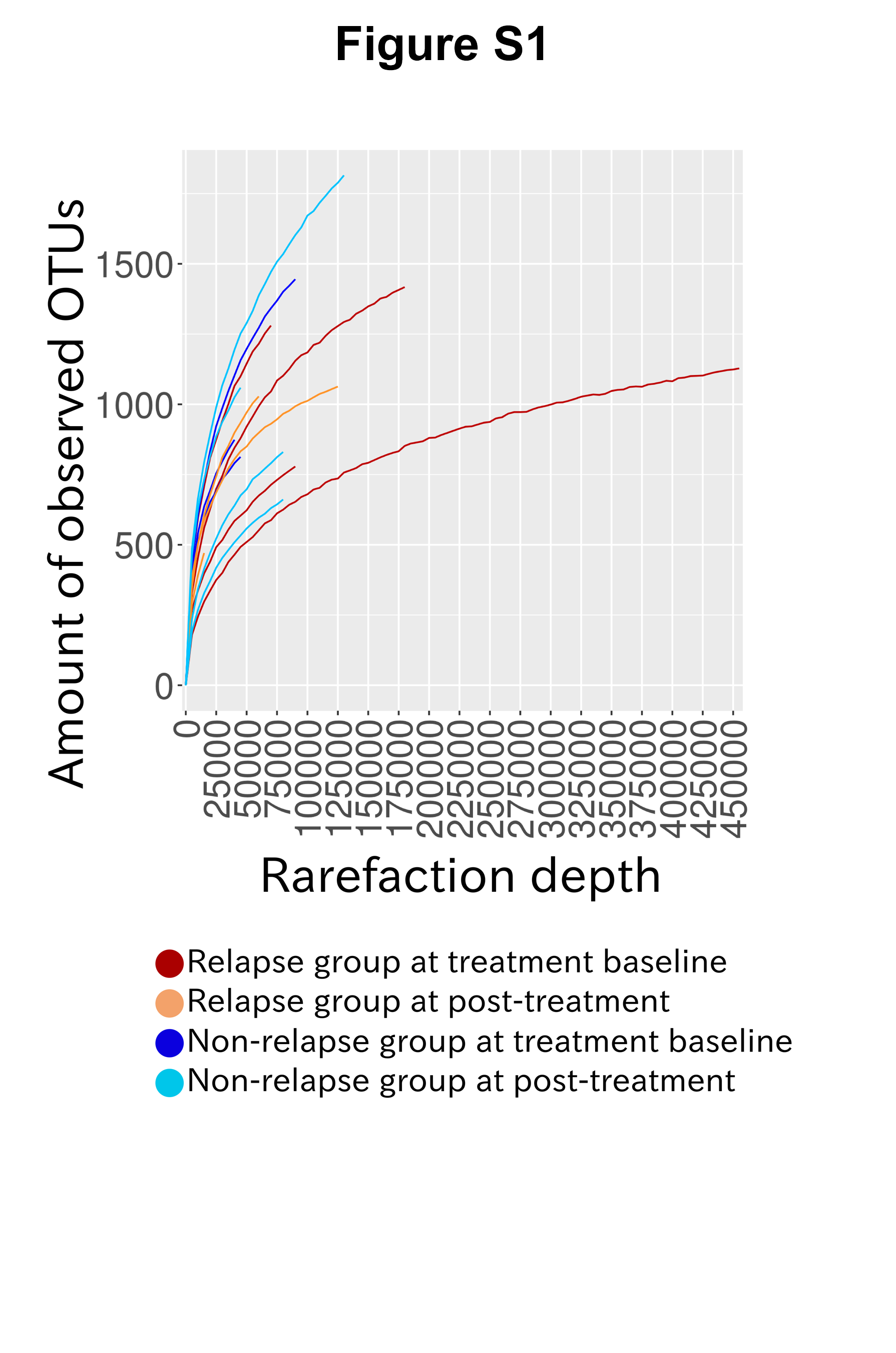

Supplement: Supplementary file 1 — Supplementary Information [file 41598_2020_76175_MOESM1_ESM.tif]

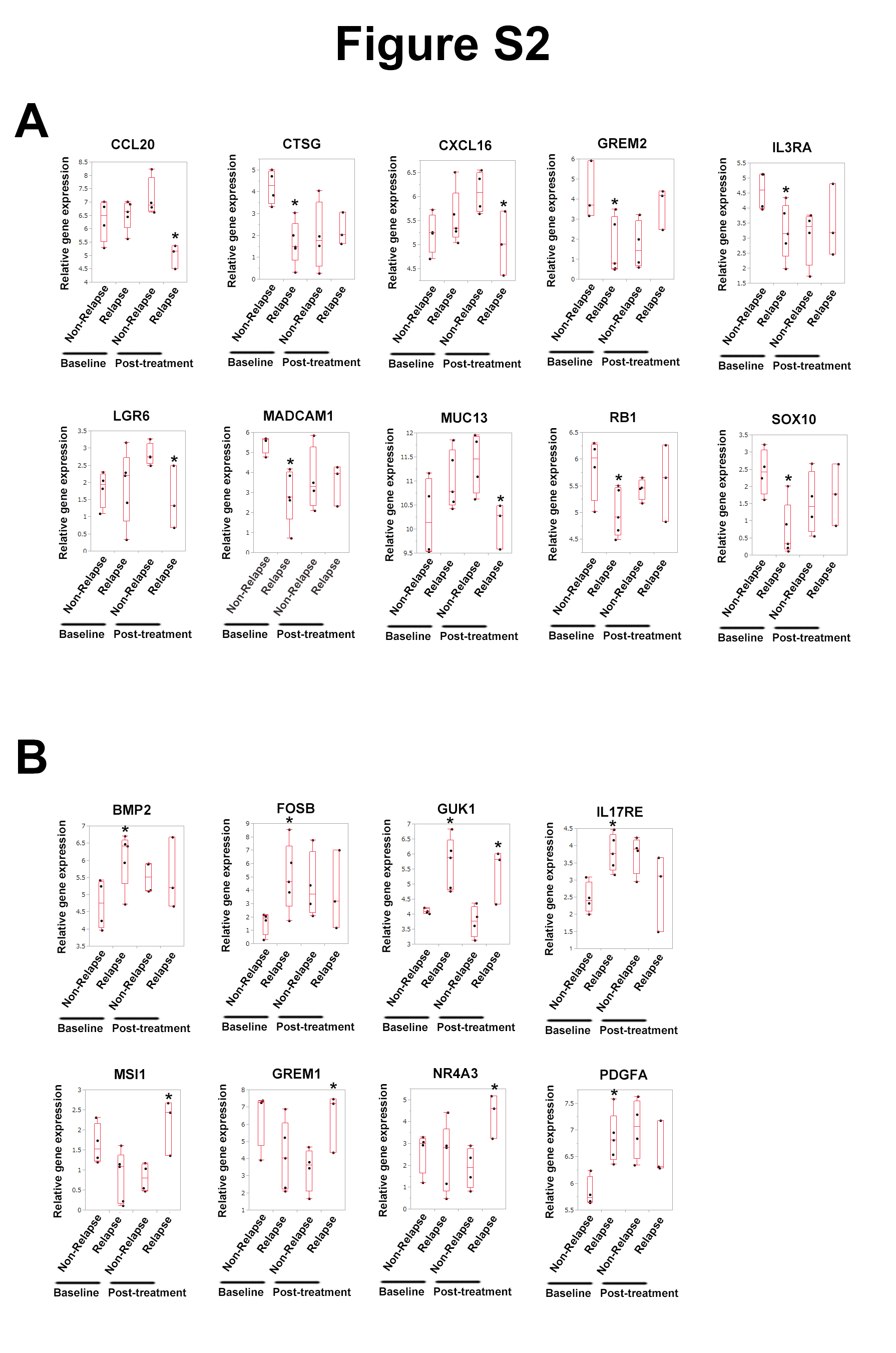

Supplement: Supplementary file 2 — Supplementary Information [file 41598_2020_76175_MOESM2_ESM.tif]

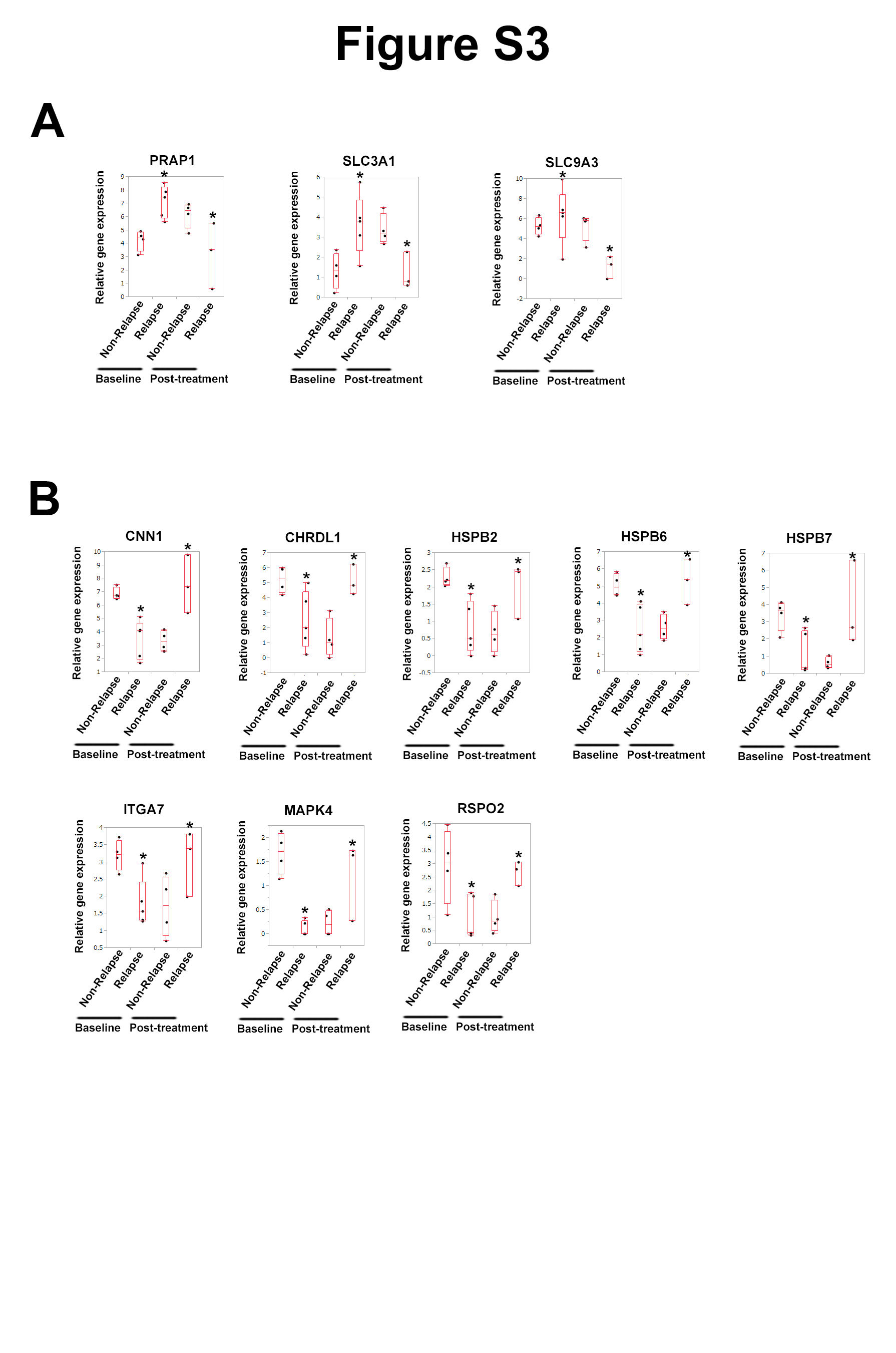

Supplement: Supplementary file 3 — Supplementary Information [file 41598_2020_76175_MOESM3_ESM.tif]

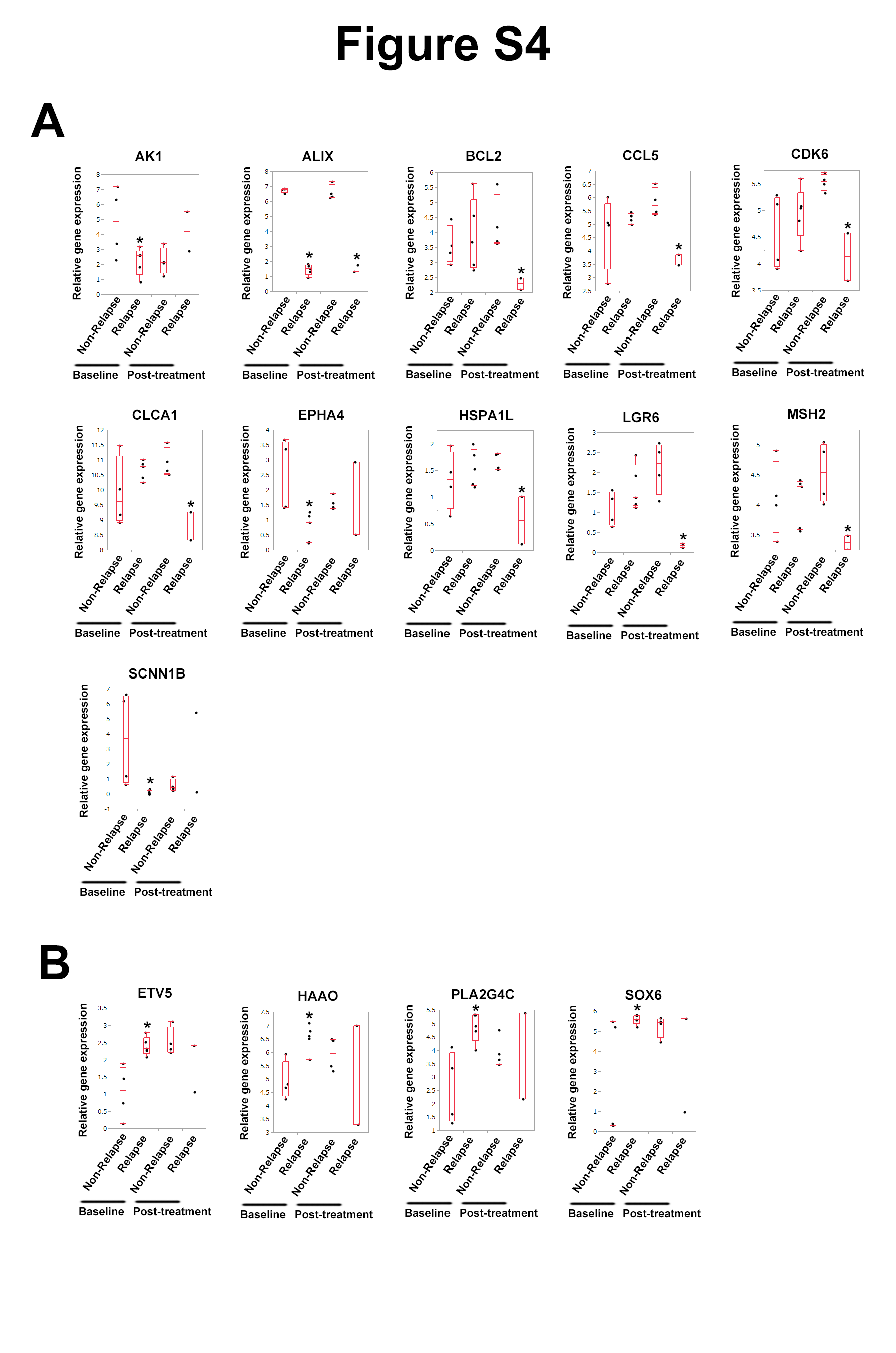

Supplement: Supplementary file 4 — Supplementary Information [file 41598_2020_76175_MOESM4_ESM.tif]

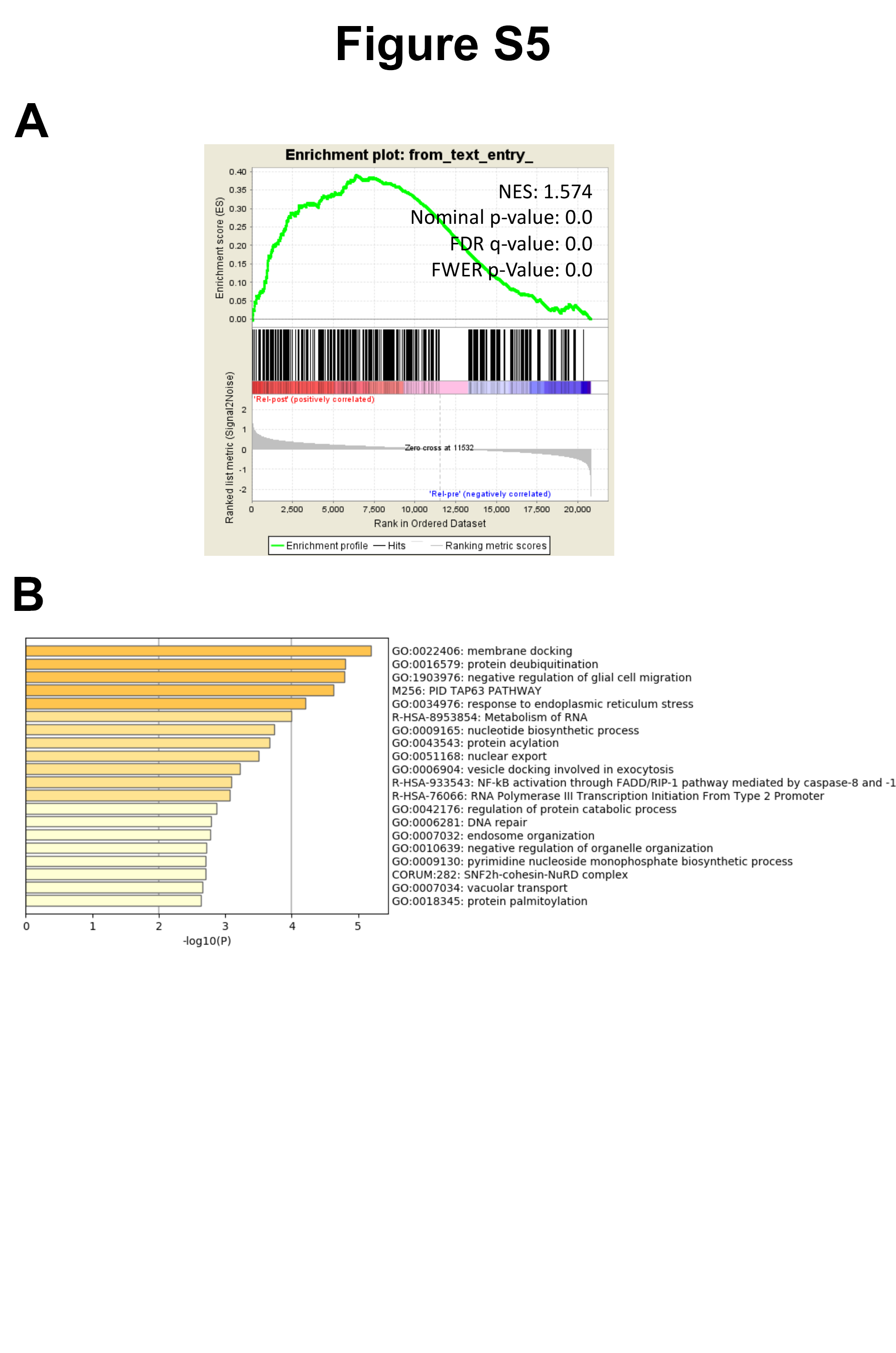

Supplement: Supplementary file 5 — Supplementary Information [file 41598_2020_76175_MOESM5_ESM.tif]

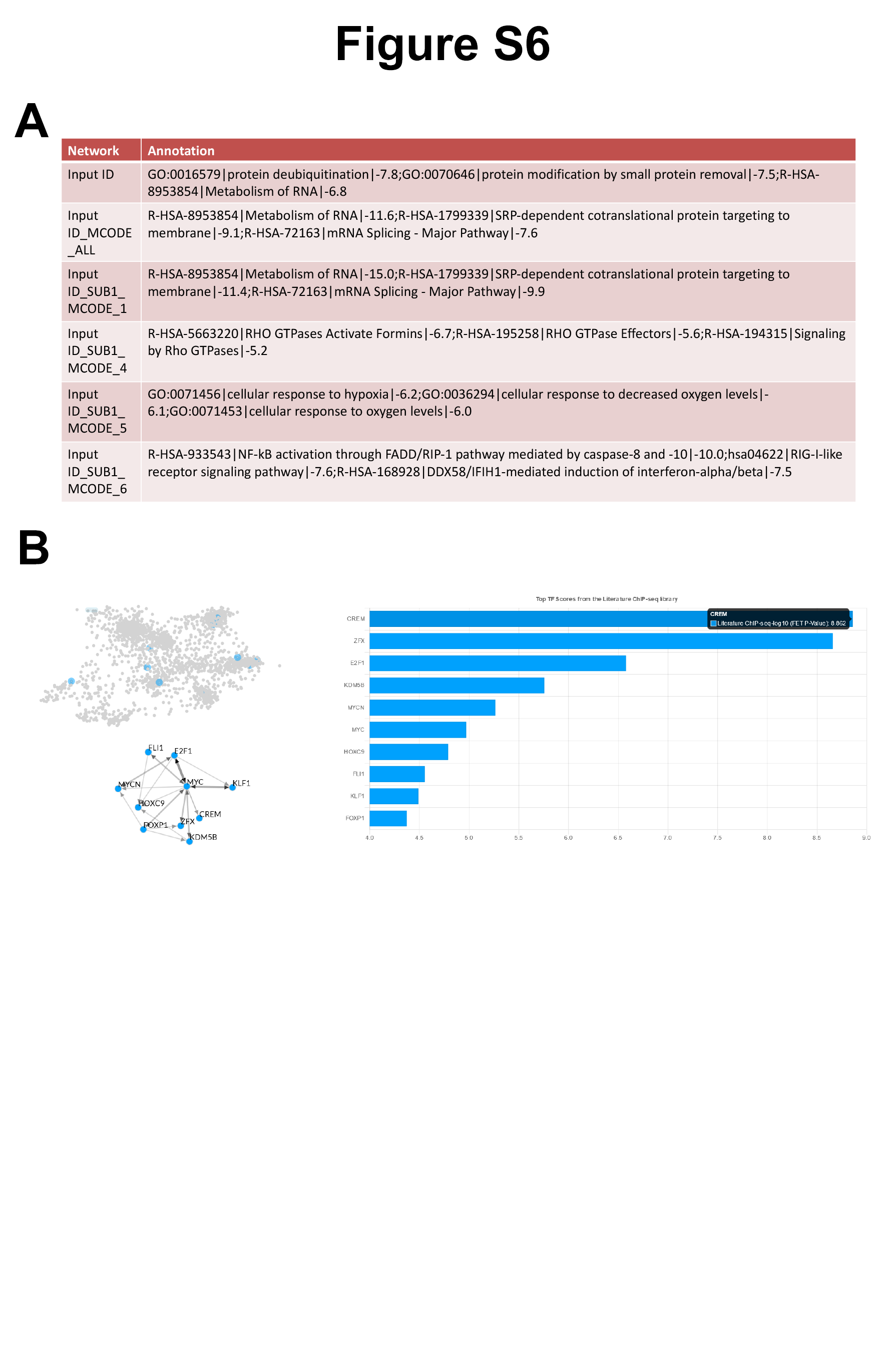

Supplement: Supplementary file 6 — Supplementary Information [file 41598_2020_76175_MOESM6_ESM.tif]

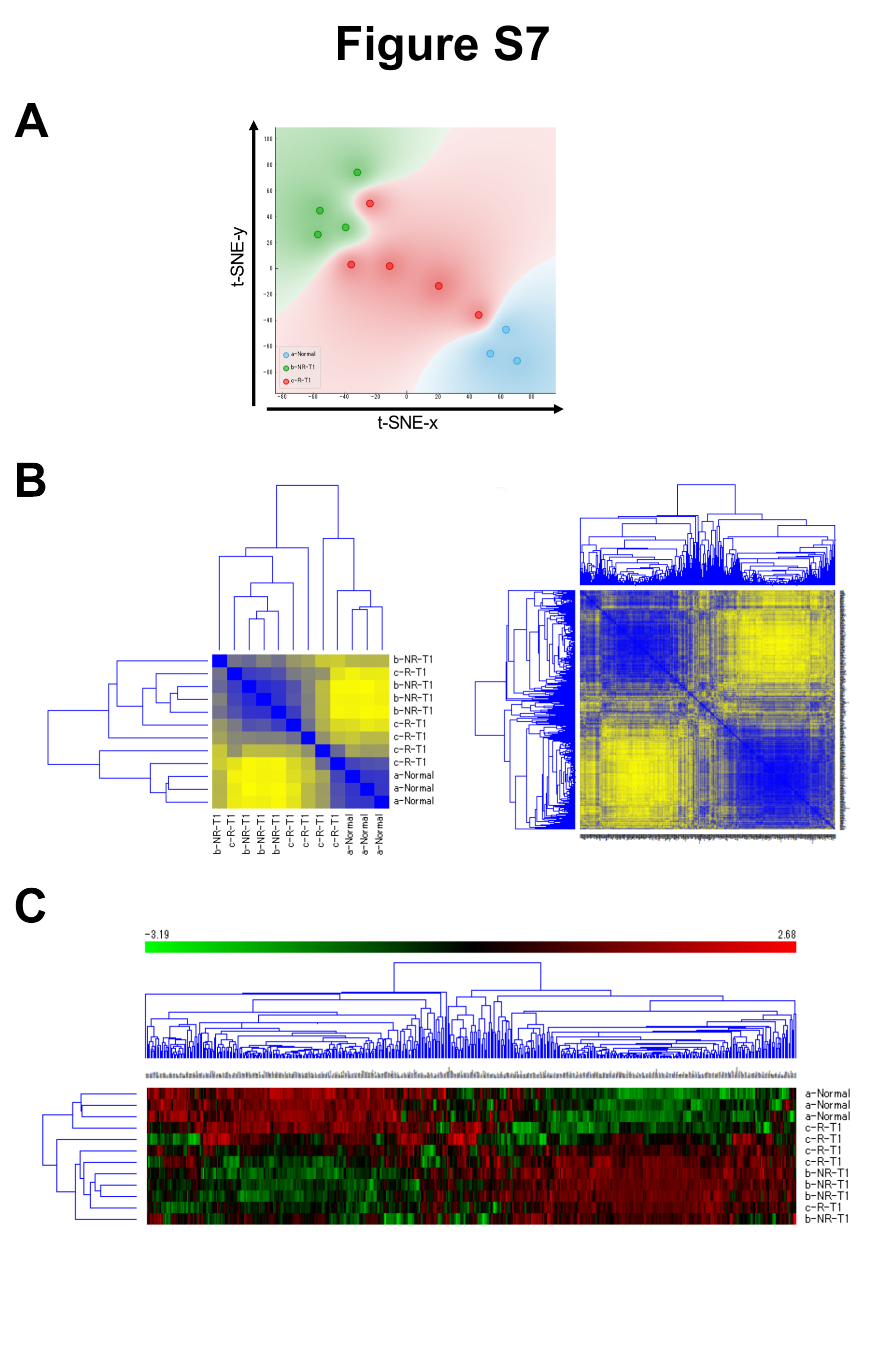

Supplement: Supplementary file 7 — Supplementary Information [file 41598_2020_76175_MOESM7_ESM.tif]

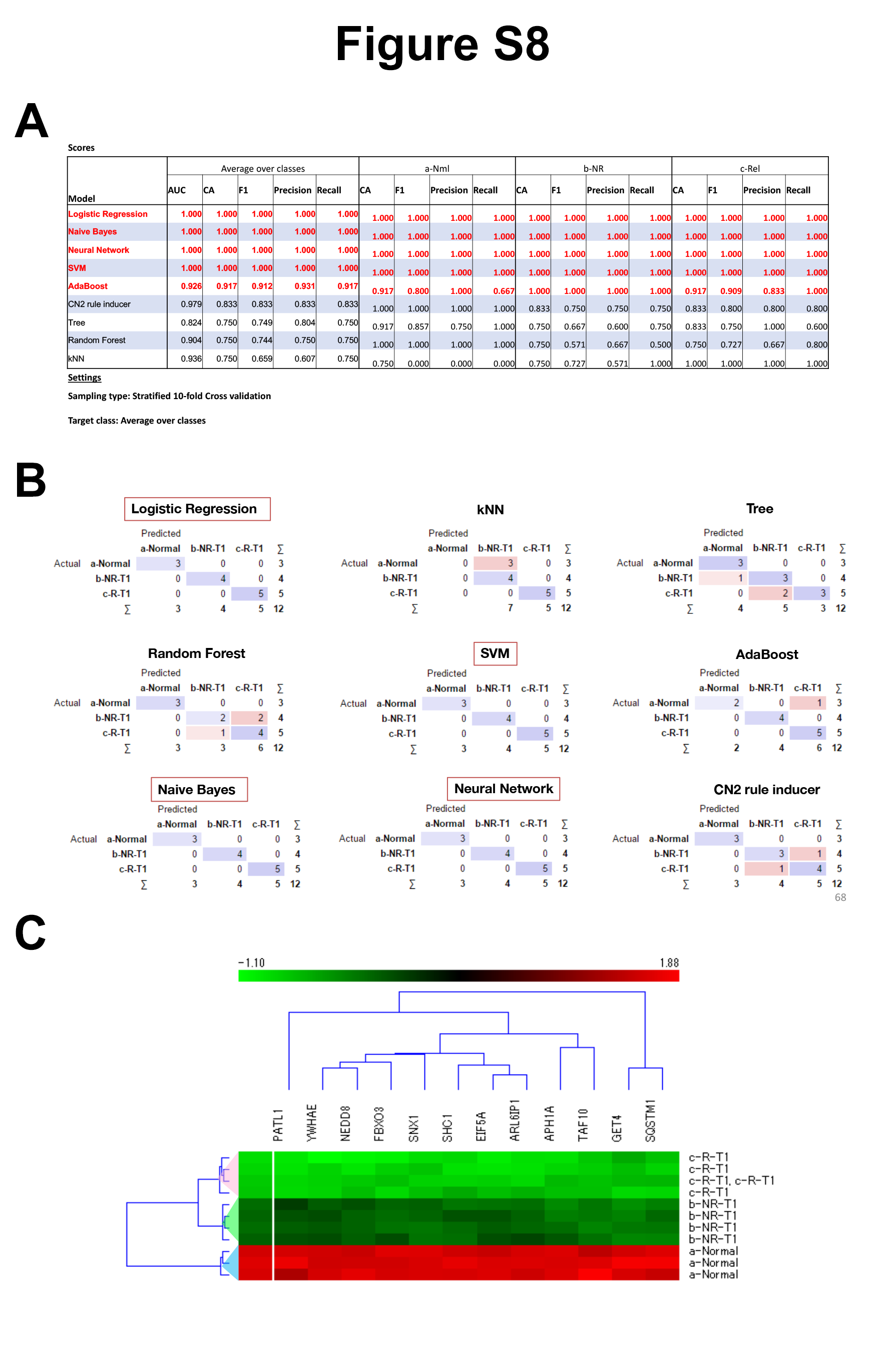

Supplement: Supplementary file 8 — Supplementary Information [file 41598_2020_76175_MOESM8_ESM.tif]
