# Supplementary material for: Mucosal microbiota and gene expression are associated with long-term remission after discontinuation of adalimumab in ulcerative colitis
Source: Sci Rep. 2020 Nov 5;10:19186. doi: 10.1038/s41598-020-76175-2 (PMC7644643; doi:10.1038/s41598-020-76175-2)
Supplement: Supplementary file 9 — Supplementary Information [file 41598_2020_76175_MOESM9_ESM.docx]

**Supplemental methods**

**Mucosal microbiota and gene expression are associated with long-term remission after discontinuation of adalimumab in ulcerative colitis**

Toshiharu Sakurai,^1*†^ Hiroki Nishiyama,^2*^ Kazuko Sakai,^3^ Marco A De Velasco,^3^ Tomoyuki Nagai,^1^ Yoriaki Komeda,^1^ Hiroshi Kashida,^1^ Akiyoshi Okada,^4^ Isao Kawai,^5^ Kazuto Nishio,^3^ Hiroyuki Ogata,^2†^ Masatoshi Kudo^1^

Short title: Prediction of long-term remission in ulcerative colitis using machine learning.

^1^Department of Gastroenterology and Hepatology, Kindai University Faculty of Medicine, 377-2 Osaka-Sayama, Osaka 589-8511, Japan,

^2^Institute for Chemical Research, Kyoto University, Uji 611-0011, Japan

^3^Department of Genome Biology, Kindai University Faculty of Medicine, Japan,

^4^Wakakusa Daiich Hospital, Higashi-Osaka, Japan

^5^Ootori Stomach and Intestine Hospital, Sakai, Japan

***Clustering 16S rRNA amplicon sequences into OTUs***

Trimming of low-quality and primer regions from paired-end reads were conducted with Trimmomatic (version 0.35) (PE, SLIDINGWINDOW:40:15, MINLEN:50) (Bolger *et al.*, 2014) and Cutadapt (version 1.11) (-e 0.18, -pair-filter = both) (Martin 2011), respectively. The paired-end reads were then aligned to form merged reads (referred to as “reads” hereafter) with FLASH (version 1.2.11) (-m 30, -M 271, -x 0.25) (Magoč and Salzberg, 2011). These reads were clustered with the representative 16S rRNA sequences of Greengenes database (version 13_8, 99_otus.fasta) (McDonald *et al.*, 2012) to form OTUs at 99% identity with UCLUST (Edgar, 2010) implemented in Quantitative Insights Into Microbial Ecology’s (QIIME version 1.9.1) (Caporaso *et al.*, 2010) parallel_pick_otus_uclust_ref.py (--max_rejects 0, --enable_rev_strand_match). A taxonomy was assigned to each OTU based on ‘99_otu_taxonomy.txt’ in Greengenes database. Chimera detection was performed on unclustred reads using uchime algorithms (Edgar *et al.*, 2011) implemented in VSEARCH (version 2.10.4), (--uchime_denovo and --uchime_ref, --db 99_otus.fasta) (Rognes *et al.*, 2016). Non-chimeric reads were clustered into additional OTUs at 99% identity using USEARCH (Edgar *et al.*, 2010) implemented in QIIME’s pick_otus.py (--otu_picking_method usearch61, --enable_rev_strand_match). Singletons and doubletons were discarded from these additional OTUs. Taxonomy was assigned to the most abundant sequence of each of these OTUs using the RDP classifier (Wang *et al.*, 2007) in QIIME’s parallel_assign_taxonomy_rdp.py (--confidence 0.8, --id_to_taxonomy_fp 99_otu_taxonomy.txt, --reference_seqs_fp 99_otus.fasta).

***Diversity analyses and differential abundance tests on 16S rRNA OTUs***

Rarefaction curves were generated with QIIME’s parallel_multiple_rarefactions.py. Specifically, reads were randomly sampled from each sample 10 times across different sampling depths with intervals of 5,000 reads. Then the average number of OTUs at each sampling depth was calculated and plotted. OTU richness and Shannon diversity index (Shannon, 1948) values were measured on randomly sampled reads per sample with QIIME’s alpha_rarefaction.py. The read sampling depth was set at the smallest sample size. For each sample, these measurements were repeated 10 times and the average value was used for comparison with the non-parametric two-sample t-test in QIIME’s compare_alpha_diversity.py with 10,000 Monte Carlo permutations.

Analysis of beta diversity with weighted UniFrac (Lozupone and Knight, 2005) distance was conducted on randomly sub-sampled reads from each sample using QIIME’s beta_diveristy.py. The read sampling depth was set at the smallest sample size. The phylogenetic tree used to measure weighted UniFrac distance was created by applying SSU-ALIGN (version 0.1.1) (Nawrocki *et al.*, 2009) and FastTree (Price *et al.*, 2010) in QIIME’s make_phylogeny.py on representative reads (i.e. the most abundant read in each OTU). QIIME’s principal_coordinates.py was used to conduct principal coordinate analysis on weighted UniFrac distance.

Significance of difference in intestinal microbial composition between two groups was tested by Adonis test (Anderson, 2001) implemented in QIIME’s compare_categories.py with 5,000 permutations. Because of weighted UniFrac distance’s sensitivity to subsampling, the process of subsampling, measuring weighted UniFrac distances and calculating Adonis P-value was repeated 10 times. The averages of the P-values were reported.

Prior to testing the significance of differential abundance of OTUs, low abundance OTUs were removed with QIIME’s filter_otus_from_otu_table.py. Specifically, OTUs with less than 100 reads from samples in comparison were omitted. In addition, OTUs present in less than 25% of the samples under comparison were also omitted. The differential abundance of OTUs between sample groups were tested for statistical significance with DESeq2’s negative binomial Wald test (Love *et al.*, 2014) implemented in QIIME’s differential_abundance.py. Within the script, alpha parameter for the “results” function in DESeq2 was set to 0.05. For comparisons of paired samples (i.e. comparisons between time points), the information of sample pairs was given to DESeq2. In this case, non-paired samples were removed from these comparisons. Benjamini-Hochberg correction was used to calculate false discovery rate (FDR) and the threshold was set at 0.05.

To further minimize the inaccurate identification of differentially abundant OTUs, those which passed the FDR threshold was further examined. For each of these OTUs, presence was checked in the samples in the high-abundance group. If any of the samples contained no reads for the OTU, we did not consider this OTU to be differentially represented. In addition, if the highest relative abundance of an OTU in the low-abundance group was larger than its relative abundances in the samples from the high-abundance group for more than half of the cases, the OTU was not considered to be differentially represented.

***Transcriptome analysis***

Pooled libraries were subjected to the Ion Chef^TM^ System (Thermo Fisher Scientific) for template preparation. Libraries were then loaded onto an Ion 550^TM^ chip and sequenced with the Ion S5^TM^ sequencing system. The Ion Torrent Suite v5.10 software was used to map read. Raw read-count data files were converted to RPKM (reads per kilobase per million reads) for read-count normalization. Differential gene expression (DGE) analysis was performed by Transcriptome Analysis Console (TAC) software (Thermo Fisher Scientific) with fold-change differences > 2.0 or < -2.0. Statistical tests were performed using ANOVA with a *P* value <0.05 as the significance cut-off, unless otherwise stated.

***Supervised machine learning analysis***

Machine learning was carried using Orange an open-source data mining suite (Demsar *et al*., 2013). Data filtering was performed to remove infrequent and overly frequent genes and median normalization was applied. A supervised approach was used to select the top 500 variable genes based on dispersion. Distributed Stochastic Neighbor Embedding (t-SNE), hierarchical clustering, correlation distance maps and FreeViz (vector-based projection) were generated in Orange and used for data visualization.

***Gene ontology and functional analysis***

Functional analysis of canonical pathways of differentially expressed genes was performed with Ingenuity Pathways Analysis (IPA) Software (Ingenuity Systems; [www.ingenuity.com](http://www.ingenuity.com)). Functional analysis of informative genes selected by machine learning were analyzed using Metascaspe (Zhou *et al.*, 2019). For gene ontology (GO) enrichment analysis, we first identified all statistically enriched terms, accumulative hypergeometric *P* values and enrichment factors were calculated and used for filtering. Remaining significant terms were then hierarchically clustered into a tree based on Kappa-statistical similarities among their gene memberships. We then selected a subset of representative terms from this cluster and convert them into a network layout. Terms with a similarity score > 0.3 are linked by an edge (the thickness of the edge represents the similarity score). The network is visualized with Cytoscape (v3.1.2). Then 0.3 kappa score was applied as the threshold to cast the tree into term clusters. The Molecular Complex Detection (MCODE) algorithm was then applied to this network to identify neighborhoods where proteins are densely connected. GO enrichment analysis was applied to each MCODE network to assign “meanings” to the network component. Gene set enrichment analysis (GSEA) was performed using GSEA 2.2.2 (Broad Institute, Cambridge, MA) (Subramanian *et al*., 2005). Gene-transcription factors interaction analysis was performed to identify upstream transcription factors of differentially expressed genes using the ChiP-X enrichment analysis version 3 (ChEA3) web application (Keenan *et al*., 2019) and the identified transcription factors were connected using known protein interactions to construct a protein interaction subnetwork.

**Reference**

Anderson M.J. A new method for non-parametric multivariate analysis of variance. Austral Ecol. 2001;26:32–46.

Bolger AM, Lohse M, Usadel B. Trimmomatic: a flexible trimmer for Illumina sequence data. Bioinformatics. 2014;30:2114-20.

Caporaso JG, Kuczynski J, Stombaugh J, Bittinger K, Bushman FD, Costello EK, et al. QIIME allows analysis of high-throughput community sequencing data. Nat Methods. 2010;7:335-6.

Demsar J, Curk T, Erjavec A, et al. Orange: Data Mining Toolbox in Python. J Mach Learn Res. 2013;14:2349−2353.

Edgar RC. Search and clustering orders of magnitude faster than BLAST. Bioinformatics. 2010;26:2460-1.

Edgar RC, Haas BJ, Clemente JC, Quince C, Knight R. UCHIME improves sensitivity and speed of chimera detection. Bioinformatics. 2011;27:2194-200.

Keenan AB, Torre D, Lachmann A, et al. ChEA3: transcription factor enrichment analysis by orthogonal omics integration. Nucleic Acids Research. 2019;47:W212-W224.

Love MI, Huber W, Anders S. Moderated estimation of fold change and dispersion for RNA-seq data with DESeq2. Genome Biol. 2014;15:550.

Lozupone C, Knight R. UniFrac: a New Phylogenetic Method for Comparing Microbial Communities. Appl Environ Microbiol. 2005;71:8228-35.

Magoč T, Salzberg SL. FLASH: fast length adjustment of short reads to improve genome assemblies. Bioinformatics. 2011;27:2957-63.

Martin M. Cutadapt Removes Adapter Sequences From High-Throughput Sequencing Reads. EMBnet.J. 2011; 17:10-2.

McDonald D, Price MN, Goodrich J, Nawrocki EP, DeSantis TZ, Probst A, et al. An improved Greengenes taxonomy with explicit ranks for ecological and evolutionary analyses of bacteria and archaea. ISME J. 2012;6:610-8.

Nawrocki EP, Kolbe DL, Eddy SR. Infernal 1.0: inference of RNA alignments. Bioinformatics. 2009; 25:1335-7.

Price MN, Dehal PS, Arkin AP. FastTree 2-Approximately Maximum-Likelihood Trees for Large Alignments. Plos One. 2010; doi:10.1371/journal.pone.0009490.

Shannon CE. A mathematical theory of communication. Bell Syst Tech J. 1948;27:379-423.

Rognes T, Flouri T, Nichols B, Quince C, Mahé F. VSEARCH: a versatile open source tool for metagenomics. PeerJ. 2016. doi: 10.7717/peerj.2584.

Subramanian A, Tamayo P, Mootha VK, et al. Gene set enrichment analysis: a knowledge-based approach for interpreting genome-wide expression profiles. Proc Natl Acad Sci U S A 2005;102:15545–15550.

Wang Q, Garrity GM, Tiedje JM, Cole JR. Naive Bayesian classifier for rapid assignment of rRNA sequences into the new bacterial taxonomy. Appl Environ Microbiol. 2007;73: 5261-5267.

Zhou Y, Zhou B, Pache L, et al. [Metascape provides a biologist-oriented resource for the analysis of systems-level datasets.](https://www.ncbi.nlm.nih.gov/pubmed/30944313) Nat Commun. 2019;10:1523.
