# Supplementary material for: Mucosal microbiota and gene expression are associated with long-term remission after discontinuation of adalimumab in ulcerative colitis
Source: Sci Rep. 2020 Nov 5;10:19186. doi: 10.1038/s41598-020-76175-2 (PMC7644643; doi:10.1038/s41598-020-76175-2)
Supplement: Supplementary file 10 — Supplementary Information [file 41598_2020_76175_MOESM10_ESM.docx]

**Supplementary Figure Legends**

**Mucosal microbiota and gene expression are associated with long-term remission after discontinuation of adalimumab in ulcerative colitis**

Toshiharu Sakurai,^1*†^ Hiroki Nishiyama,^2*^ Kazuko Sakai,^3^ Marco A De Velasco,^3^ Tomoyuki Nagai,^1^ Yoriaki Komeda,^1^ Hiroshi Kashida,^1^ Akiyoshi Okada,^4^ Isao Kawai,^5^ Kazuto Nishio,^3^ Hiroyuki Ogata,^2†^ Masatoshi Kudo^1^

Short title: Prediction of long-term remission in ulcerative colitis using machine learning.

^1^Department of Gastroenterology and Hepatology, Kindai University Faculty of Medicine, 377-2 Osaka-Sayama, Osaka 589-8511, Japan,

^2^Institute for Chemical Research, Kyoto University, Uji 611-0011, Japan

^3^Department of Genome Biology, Kindai University Faculty of Medicine, Japan,

^4^Wakakusa Daiich Hospital, Higashi-Osaka, Japan

^5^Ootori Stomach and Intestine Hospital, Sakai, Japan

Figure S1. The amount of OTUs observed at each rarefaction depth. The color codes for the samples are red: relapse group at treatment baseline, orange: relapse group at post-treatment, blue: non-relapse group at treatment baseline, turquoise: non-relapse group at post-treatment.

Figure S2. Gene expression signatures in inflamed mucosae (rectum) between the relapse group (Relapse) and non-relapse group (Non-relapse). (A) Upregulated genes in the non-relapse group are shown compared with those in the relapse group. (B) Downregulated genes in non-relapse group are shown compared with those in the relapse group. * *P* < 0.05 compared with the non-relapse group at treatment baseline (Baseline) or at post-treatment.

Figure S3. Gene expression signatures in inflamed mucosae (rectum) between the relapse group (Relapse) and the non-relapse group (Non-relapse). (A) Genes that were downregulated at treatment baseline (Baseline) and upregulated at post-treatment (Post-treatment) in the non-relapse group compared with those in the relapse group are shown. (B) Genes that were upregulated at Baseline and downregulated at Post-treatment in the non-relapse group compared with those in the relapse group are shown. * *P* < 0.05 compared with the non-relapse group at Baseline and Post-treatment.

Figure S4. Gene expression signatures in non-inflamed mucosae (ileum or right colon) between the relapse group (Relapse) and the non-relapse group (Non-relapse). (A) Upregulated genes in the non-relapse group are shown compared with those in the relapse group. (B) Downregulated genes in the non-relapse group are shown compared with those in the relapse group. * *P* < 0.05 compared with the non-relapse group at treatment baseline (Baseline) or at post-treatment (Post-treatment).

Figure S5. (A) Gene set enrichment analysis (GSEA) of genes enriched in relapsed UC patients versus baseline. (B) Bar plot of the top 20 enriched ontology clusters.

Figure S6 (A) Summary of statistically significant MCODE network clusters and their biological significance. (B) Bar chart, global and local networks corresponding to the top ten transcription factors mined from the literature using the ChEA3 library.

Figure S7 Unsupervised clustering analysis between the transcriptome of normal colon, and UC patients (relapsed and non-relapsed) at baseline. (A) t-SNE visualization based 500 genes extracted based on dispersion without group assignments. Corresponding Pearson correlation matrix heatmap showing the distance between patient classes (B) and selected 500 genes (C). Clustering analysis of 500 genes selected. Heatmap shows unsupervised hierarchical clustering using average linkage and Euclidean distance.

Figure S8 (A, B) Summary table (A) of cross-validation accuracy estimation and corresponding confusion matrix (B). (C) Hierarchical clustering of top 12 relevant features. Heatmap shows unsupervised hierarchical clustering using average linkage and Euclidean distance.
