# Supplementary material for: Mucosal microbiota and gene expression are associated with long-term remission after discontinuation of adalimumab in ulcerative colitis
Source: Sci Rep. 2020 Nov 5;10:19186. doi: 10.1038/s41598-020-76175-2 (PMC7644643; doi:10.1038/s41598-020-76175-2)
Supplement: Supplementary file 11 — Supplementary Information [file 41598_2020_76175_MOESM11_ESM.docx]

**Supplementary Table 1. Genes differentially expressed between the relapse and the non-relapse groups**

|  | Genes upregulated in **Non-relapse** group | | Genes upregulated in **Relapse** group | |
| --- | --- | --- | --- | --- |
|  | **Uninflamed** mucosae | **Inflamed** mucosae | **Uninflamed** mucosae | **Inflamed** mucosae |
| **Week 0** | ALIX, AK1, EPHA4, SCNN1B | ALIX, CNN1, CTSG, GREM2, HSPB2, HSPB4, HSPB7, ITGA7, IL3RA, MADCAM1, MAPK4, RB1, RSPO2, SOX10. | ETV5, HAAO, PLA2G4C, SOX6 | BMP2, CNN1, FOSB, GUK1, IL17RE, IL22RA, PDGFA, PRAP1, SLC3A1, SLC9A3. |
| **Week 24** | ALIX, BCL2, LGR6, CCL5, CDK6, CLCA1, HSPA1L, MSH2 | ALIX, FCGBP, CCL20, CXCL16, IL22RA, LGR6, MUC13, PRAP1, SLC3A1, SLC9A3. | AIFM3, BNIP3, IRS1 | CHRDL, CNN1, GREM1, GUK1, HSPB2, HSPB6, HSPB7, ITGA7, MAPK4, MSI1, NR4A3, RSPO2. |
